# Analysis of informed consent forms of patients undergoing cancer genetic testing in the era of next-generation sequencing

**DOI:** 10.1186/s13053-025-00309-8

**Published:** 2025-02-21

**Authors:** Tina Kerševan, Tina Kogovšek, Ana Blatnik, Mateja Krajc

**Affiliations:** 1 Department of Clinical Cancer Genetics, Institute of Oncology, Zaloška cesta 2, Ljubljana, 1000 Slovenia; 2 Faculty of Arts, University of Ljubljana, Aškerčeva cesta 2, Ljubljana, 1000 Slovenia; 3 Faculty of Social Sciences, University of Ljubljana, Kardeljeva pl. 5, Ljubljana, 1000 Slovenia; 4 Faculty of Medicine, University of Ljubljana, Korytkova 2, Ljubljana, 1000 Slovenia; 5 Faculty of Health Sciences, University of Primorska, Polje 42, Izola, 6310 Slovenia

**Keywords:** Informed consent, Consent, Genetic counselling, Genetic testing

## Abstract

**Background:**

The Department of Clinical Cancer Genetics at the Institute of Oncology Ljubljana offers genetic counselling and testing to cancer patients and their relatives. Before undergoing genetic testing, patients sign the informed consent form. In addition to giving consent for collection of biological material and genetic testing, patients decide about storage of biological material and participation in international databases. Furthermore, patients decide whether the information regarding their test results may be revealed to their blood relatives and whether they want to be informed about secondary findings.

**Methods:**

Using the signed consent forms, we investigated the effect of selected factors on patients’ decisions. Using different statistical methods, we tried to determine the proportion of patients who opted for different items and the effect of gender, age and cancer diagnoses on their decisions.

**Results:**

Nearly all (99.6%) patients, regardless of gender, age, and presence of oncological diagnosis, consented to the storage of their biological material, 98.4% of patients, regardless of gender, age, and presence of oncological diagnosis, wanted to be included in international databases in a pseudo-anonymised form, 98.8% of patients, irrespective of gender, age, and presence of oncological diagnosis, allowed blood relatives to see their results, and 98.4% of patients, irrespective of gender, age and presence of oncological diagnosis, wanted to know whether secondary findings were detected when genetic analysis of their biological material was performed. Men are, on average, more likely to consent but the difference between genders is not statistically significant. Patients without oncological disease were more likely to agree to be included in international databases than patients with a confirmed oncological diagnosis.

**Conclusions:**

Our results show that the vast majority of patients were in favour of the options they were offered. Most importantly, the majority of them allow their genetic test results be revealed to their blood relatives when needed and would participate in international databases. Research in rare diseases, including rare cancer genetic predisposition syndromes, is crucial for optimal diagnostic, prevention and treatment options for patients with rare genetic disorders. The results are also important for refining the approach to pre-and post-test cancer genetic counselling.

## Background

Cancer arises through an accumulation of genetic or epigenetic alterations affecting genes that play an essential role in the transformation of normal cells to cancer cells. The majority of cancers are sporadic. They are caused by genetic alterations in a certain tissue (i.e. somatic alterations) and are very likely induced by environmental factors [1]. Conversely, up to 10% of cancers occur due to the inherited pathogenic variant in cancer predisposition genes. Individuals who carry such a pathogenic variant have an increased risk of developing certain types of cancer. Based on their genetic testing results, they can profit from prevention strategies, e.g. high-risk cancer screeningor risk-reducing surgeries. Furthermore, by knowing their genetic status, cancer patients may benefit from personalised cancer treatment. When a pathogenic variant is detected in an individual, blood relatives may opt for genetic counselling and testing.

Cancer genetic counselling and testing was implemented in Slovenia soon after the discovery of *BRCA1* and *BRCA2* genes. In 1999, the Cancer Genetics Clinic was established in a multidisciplinary setting at the Institute of Oncology Ljubljana (IOL), the principal national institution offering comprehensive cancer care. The Institute is a tertiary referral centre and provides cancer genetic counselling, genetic screening, testing, and surveillance of high-risk individuals.

Before undergoing genetic testing, patients must attend genetic counselling. They sign an informed consent form, agreeing to genetic testing and acknowledging they received all the necessary information during genetic counselling. It is always obtained before the biological sample is taken for testing (usually peripheral blood). This clinical pathway has been in place since 1999 and the content of the consent form has been amended and updated several times over the years, whenever there were new legal or scientific requirements. The changes have been mainly influenced by the implementation of new legislation, such as the adoption of the European Union General Data Protection Regulation (GDPR) and new developments in molecular diagnostic techniques. The consent form must be also signed by a treating health professional ordering the test, usually a medical doctor who is a specialist in clinical genetics. The explanatory duty of the doctor is to inform the patient about the importance of the genetic test, its benefits, risks, and limitations and its relevance for patient’s relatives, explain the possible results and their potential impact on the patient’s further treatment or surveillance.

As reported in other studies, the process of completing the consent form can be used to help individuals understand that genetic testing is entirely voluntary. Besides, it helps inform individuals of the purpose of genetic testing, their participation in further research [2], the limitations and possible consequences of the results, including possible secondary findings [3], sample storage, and their inclusion in international databases. Furthermore, it is essential that the patient fully understands the procedure, the benefits and limitations of the test, and the possible consequences of the test results before consenting to a genetic test [4]. The referring physician needs to clarify the content of the consent in a way that is understandable, and to be available for further questions and clarifications [5].

Consistent use of a standardised informed consent procedure can improve communication between clinicians and patients and increase understanding of the importance of genetic testing [6]. Pre-test genetic counselling can improve the patient’s knowledge, allow for rational autonomy and individual decision-making, and reduce anxiety, distress and depression. Reliability, openness and honesty of health professionals are essential and make the process of fulfilling the consent form easier [7].

The patient is informed, as stated on the consent form, that it is possible to withdraw the consent after signing it without any explanation and that this action will not affect their medical care.

Slovenian patients have been signing informed consent forms before undergoing cancer genetic testing since 1999, but until now, no evaluation of their decisions has been performed.

The aim of our study was therefore to analyse patients’ decisions on options provided in the informed consent form. We aimed to determine the percentage of individuals who agreed to genetic testing and consented to (i) storage of biological material, (ii) inclusion in international databases, (iii) the possibility of informing blood relatives about the result, and (iv) reporting of secondary findings. We also wanted to determine whether there were significant differences in persons’ decision-making when completing the consent form according to gender, age, and diagnosis of cancer.

### Research questions

RQ1: Are most patients in favour of the different options presented in the consent form?

RQ2: Do age, gender, and absence/presence of oncological disease affect patient decisions when completing the consent form?

### Methods

### Study cohort

We analysed 5244 consecutively obtained consent forms that were filled out before genetic testing for cancer predisposition whenever a hereditary cancer syndrome was suspected in a patient or a family. Only patients undergoing multigene panel testing using next-generation sequencing in the years 2019–2022 were included in our study. All individuals were counselled and assessed in the Department of Clinical Cancer Genetics, the IOL, Slovenia, between 2019 and 2022. Demographic and medical data (cancer status) was obtained from hospital medical records.

**Fig. 1:**
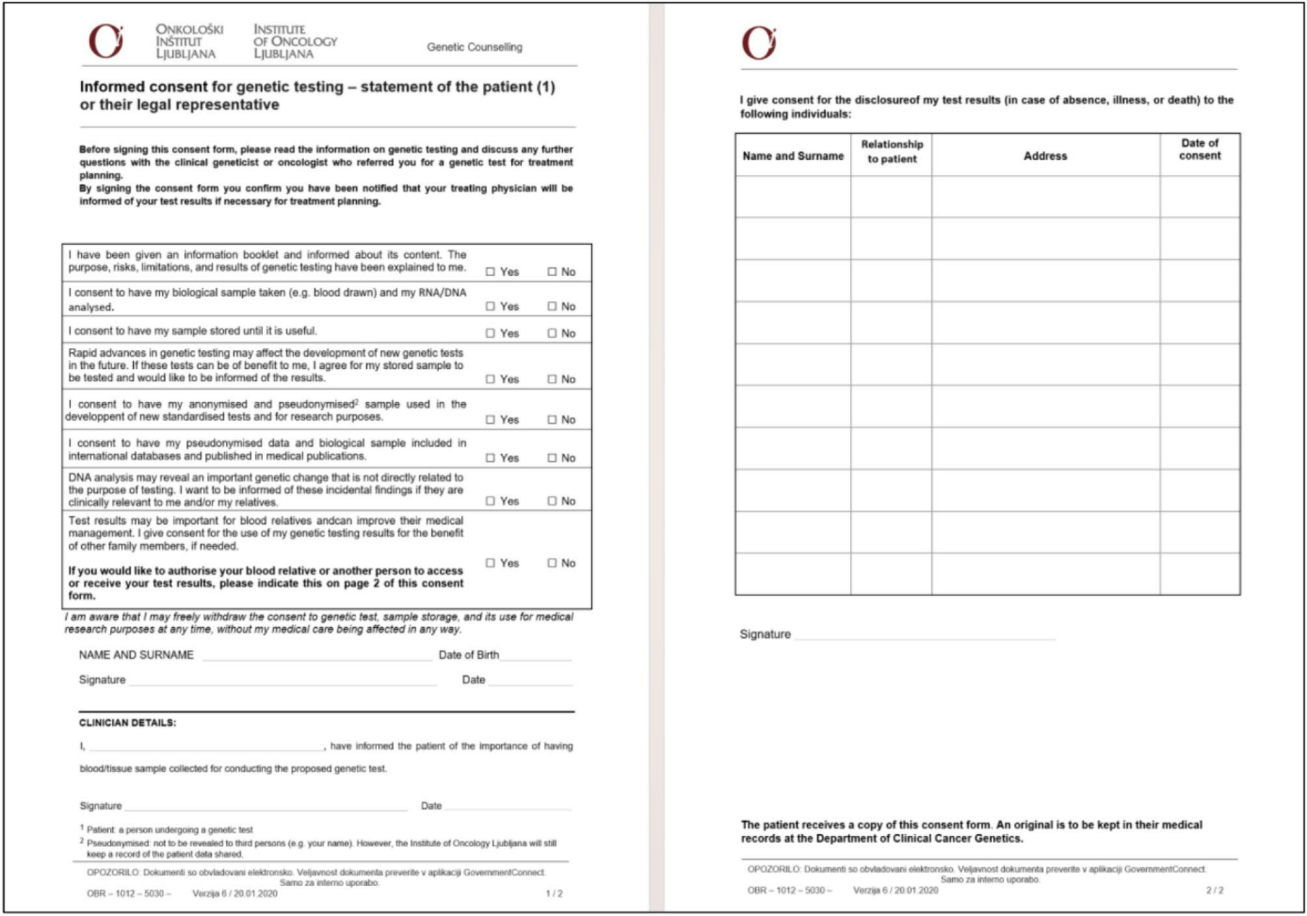
Informed consent for genetic testing

All enrolled patients voluntarily completed the consent form (Fig. 1) before genetic testing in accordance with our clinical pathway [8].

The patient in this paper is defined as every individual (with or without cancer diagnosis) referred for medical assessment to the Department of Clinical Cancer Genetics.

The consent form is always signed by a patient and medical doctor or genetic counselor. In case a tested individual is younger than 15 years old, her or his legal representative signs the consent.

### Consent form content

**Fig. 2:**
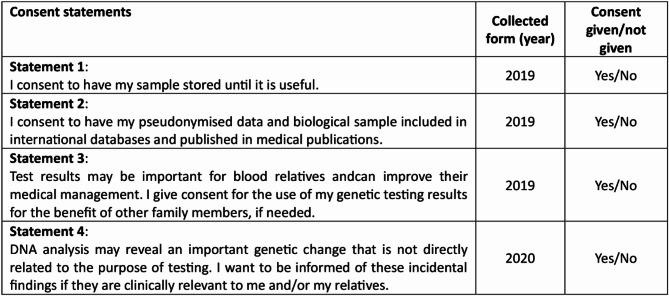
The content of consent forms

Data collected for further evaluation is presented in Fig. 2. We examined individuals’ decisions on the following topics: storage of biological material, inclusion in international databases, informing blood relatives of the result, and reporting of secondary findings.

A secondary finding is defined as a finding of a pathogenic variant in a gene unrelated to the indication for testing whichmay be relevant to the subject’s health, and may be medically actionable. Secondary findings can be detected when genetic testing with next-generation sequencing is performed, as previously described [9, 10]. The American College of Medical Genetics and Genomics (ACMG) established a Secondary Findings Maintenance Working Group in 2014, which publishes recommendations for reporting secondary findings in genomic testing. According to their research, on average, a secondary finding is reported in 1–6% of tested individuals [11]. In 2022, an updated list, i.e. “ACMG SF v3.1 list” of 78 different genes recommended for return as secondary findings was published [12].

Patients may decide and consent to storing biological material at the Department for Molecular Diagnostics. The biological material, is collected and stored permanently (or for as long as it is still usable).

Consent is also requested for the use of pseudo-anonymised personal and biological data for future research. This means that the patient permits their medical data and a sample of biological material to be further analysed in a research setting, under a pseudonym, in an anonymous form. Personal data is stored in a separate database and cannot be attributed to a specific individual.

Every patient has the right to medical data confidentiality. For this reason, each patient decides independently whether to allow or refuse to share their genetic testing results with their blood relatives when this is needed for medical reasons.

### Statistical analysis

We used descriptive statistical analysis methods (frequency distributions, basic descriptive statistics) and bivariate methods to analyse the association of variables (contingency table and chi-square test, t-test) and tested the assumption of the equality of means in all groups (analysis of variance). We examined the effect of selected factors (gender, age, and confirmed cancer diagnosis) on the consent of patients when signing the consent form.

To facilitate interpretation of the results, the values of the age variable were ranked according to the ordinal scale of measurement: 1– younger adults (up to 40 years), 2– mid-life adults (41–60 years), and 3– older adults (61 years and above).

We used the form “Informed Consent for Genetic Testing” as the measurement instrument. It consisted of seven questions in 2019 and eight questions in 2020, with the addition of a question regarding the secondary findings. Patients answered yes/no questions (Fig. 2).

## Results

### Response rate

The response rate was 100% (5244) for all questions. The number of responses is lower for secondary findings than the other variables studied because the statement regarding secondary findings was introduced in 2020. The response rate of a question on secondary findings was also 100% (3649).

### Study sample characteristics

The study included 1164 men and 4080 women, representing 22% and 78%, respectively. The average age at testing and signing the consent was 53.63 years, with a standard deviation of 15 years. The youngest patient was two years old and the oldest was 93 years old. A total of 16 underage patients were assessed. In this case, the parents or legal guardians completed and signed the consent forms.

### Stratification of the cohort

Younger adults cohort included 1066 patients (20%), mid-life adults cohort included 2288 (44%) and older adults 1890 (36%) patients.

31% of the patients included in the study did not have a cancer diagnosis, and 69% had an oncological diagnosis. The cohort of affected patients included those diagnosed with at least one cancer diagnosis as well as patients with colorectal polyposis.

### Statement content analysis: sample storage, international databases, result disclosure to relatives, secondary findings

Table 1 shows the percentage of patients agreeing to four different statements in the consent form (Fig. 2). Less than 0.5% refused to have their biological material sample to be stored (Statement 1). Regarding Statement 2, 1.6% did not give their consent for research, whereas 98.8% of patients answered “yes” to Statement 3 regarding their blood relatives.

Table 1 also shows that 3649 participants provided responses for Statement 4–98.4% consented and wanted to know about secondary findings.

**Table 1:** Responses to consents given on sample storage, participation in international databases, result disclosure to relatives, and secondary findings

		Sample storage		International databases		Result disclosure		Secondary findings	
		Frequency	Valid Percent	Frequency	Valid Percent	Frequency	Valid Percent	Frequency	Valid Percent
N and %	Does not agree	20	0.4	86	1.6	62	1.2	57	1.6
	Agree	5224	99.6	5158	98.4	5182	98.8	3592	98.4
	Total	5244	100.0	5244	100.0	5244	100.0	3649	100.0

### Analysis by gender

Among all men included in our study (1164), 99.7% consented to sample storage, and among all women, 99.6% consented to sample storage. The difference between men and women is not statistically significant.

Table 2 shows that among all men, 99.3% consented to submit biological material to international databases, and among all women, 98.1% consented. The χ^2^ value is equal to 8.42, and the association is statistically significant because the significance level is less than 0.05. Cramer’s V, however, indicates a weak association.

**Table 2:** Consent for international databases by gender

			International databases		Total
			Does not agree	Agrees	
Gender	Men	Count	8	1156	1164
		% within Gender	0.7%	99.3%	100.0%
	Women	Count	78	4002	4080
		% within Gender	1.9%	98.1%	100.0%
Total		Count	86	5158	5244
		% within Gender	1.6%	98.4%	100.0%

X^2^ = 8.42 (*p* =.004), Cramer’s V = 0.040

Among all patients, 98.7% of men and 98.8% of women allowed blood relatives to be informed about their results. However, the association between gender and consent to blood relatives’ access is not statistically significant.

Most men (98.8%) and women (98.3%) wanted to know if a secondary finding was detected during the analysis. However, the association between gender and consent to secondary findings is not statistically significant.

### Analysis by age

When stratified by age, 99.7% of mid-life adults, 99.3% of younger adults, and 99.6% of older adult patients consented to sample storage. Although the χ^2^ value is equal to 2.987, the association is not statistically significant because the significance level is greater than 0.05. Cramer’s V indicates a weak association.

Table 3 shows that 98.9% of mid-life adults, 98.2% of younger adults, and 97.8% of older adult patients agreed to be included in the international databases. The χ^2^ value is equal to 7.014; the association is statistically significant because the significance level is less than 0.05. Cramer’s V indicates a weak association.

**Table 3:** Consent for inclusion in international databases by age

			International databases		Total
			Does not agree	Agrees	
Age	Up to 40 years	Count	19	1047	1066
		% within Age	1.8%	98.2%	100.0%
	41–60 years	Count	26	2262	2288
		% within Age	1.1%	98.9%	100.0%
	Above 61 years	Count	41	1849	1890
		% within Age	2.2%	97.8%	100.0%
Total		Count	86	5158	5244
		% within Age	1.6%	98.4%	100.0%

X^2^ = 7.014 (*p* =.030), Cramer’s V = 0.037

For Statement 3, 98.9% of all mid-life adults, 98.8% of younger adults, and 98.7% of older adult patients consented to blood relatives seeing their results. The χ^2^ value is equal to 0.294; the association is not statistically significant because the significance level is greater than 5%. Cramer’s V indicates a weak association.

Table 4 shows that 99.2% of mid-life adults, 98.4% of younger adults, and 97.7% of older adult patients wanted to know about secondary findings. The χ^2^ value is equal to 11.626, and the association is statistically significant because the significance level is less than 0.05. Cramer’s V indicates a medium correlation.

**Table 4:** Consent for secondary findings by age

			Secondary findings		Total
			Does not agree	Agrees	
Age	Up to 40 years	Count	8	480	488
		% within Age	1.6%	98.4%	100.0%
	41–60 years	Count	14	1654	1668
		% within Age	0.8%	99.2%	100.0%
	Above 61 years	Count	35	1458	1493
		% within Age	2.3%	97.7%	100.0%
Total		Count	57	3592	3649
		% within Age	1.6%	98.4%	100.0%

X^2^ = 11.626 (*p* =.003), Cramer’s V = 0.056

The sum of the consents for sample storage, international databases, and relatives shows a minimally higher arithmetic mean for adults (0.001); younger and older adults were less likely to agree than adults. However, the association is not statistically significant because the significance level is greater than 0.05.

### Analysis stratified by affected and non-affected patients

Among all patients diagnosed with an oncological disease, 99.7% consented to sample storage, and among all patients without an oncological disease, 99.5% consented. The value of χ^2^ is equal to 1.72. The association is not statistically significant because the significance level is greater than 0.05.

Table 5 shows that 98.9% of patients without an oncological diagnosis agreed to be included in the international databases, while 98.1% of patients with an oncological diagnosis agreed to be included in the international databases. The χ^2^ value is equal to 4.44. The association is statistically significant because the significance level is less than 0.05. Cramer’s V indicates a weak association.

**Table 5:** Consent for international databases by presence of oncological disease

			International databases		Total
			Does not agree	Agrees	
Diagnosis when signing consent	No	Count	18	1628	1646
		% within Diagnosis when signing the consent	1.1%	98.9%	100.0%
	Yes	Count	68	3530	3598
		% within Diagnosis when signing the consent	1.9%	98.1%	100.0%
Total		Count	86	5158	5244
		% within Diagnosis when signing the consent	1.6%	98.4%	100.0%

X^2^ = 4.440 (*p* =.035), Cramer’s V = 0.029

Of all patients diagnosed with cancer, 98.9% agreed to allow their results to be shared with blood relatives. Among patients without oncological disease, 98.6% did so. The χ^2^ value is equal to 0.95. The association is not statistically significant because the significance level is greater than 0.05. Cramer’s V indicates a weak association.

Among all patients diagnosed with cancer, 98.3% wanted to know about secondary findings. Among patients without cancer, that percentage was 98.9%. The χ^2^ value is equal to 1.06, and the association is not statistically significant because the significance level is greater than 0.05. Cramer’s V indicates a weak association.

Next, we aggregated responses to statements regarding sample storage, inclusion in international databases, and access to blood relatives (all “yes” answers for each respondent). Finally, we checked whether there were differences between the patients’ averages for these responses. The opt-in option for secondary findings was excluded and analysed separately. Men are on average slightly more likely to consent (2.98), while the mean for women is 2.96. A t-test for independent samples shows that the difference between the means of men and women is not statistically significant (t = 1.840, *p* >.05).

Table 6 shows that men are slightly more likely to favour consent (2.98 for men and 2.96 for women).

**Table 6:** T-test by gender

	Gender	*N*	Mean	Std. Deviation	Std. Error Mean
Sum of consents	Men	1164	2.9768	0.17192	0.00504
	Women	4080	2.9654	0.22805	0.00357

t = 1.840 (*p* =.066)

Next, we tested whether there were statistically significant differences in the average number of favourable responses depending on the patients’ age and the presence of cancer. As there are three age groups, we used the analysis of variance method. The minimally higher value of the arithmetic mean (0.02) for mid-life adults has shown that mid-life adults are more likely to consent than younger and older adults. However, the dispersion within groups did not stand out, and the association is not statistically significant.

A t-test for equality of arithmetic means has shown that the differences in the arithmetic means were not statistically significant for the number of favourable responses and the presence of an oncological diagnosis. Patients with cancer diagnoses were slightly less likely to consent, but the difference is not statistically significant.

## Discussion

Our study was the first to analyse responses of patients giving informed consent for genetic testing in Slovenia. Using frequency distribution, we have shown that more than 98% of patients, regardless of gender, age, and presence of oncological diagnosis, consented to the storage of their biological material, wanted to be included in international databases in a pseudo-anonymised form, allowed blood relatives to see their results, and wanted to know whether secondary findings were detected in the analysis of their biological material.

Men are, on average, more likely to consent but the difference is not statistically significant. A minimally higher value for the arithmetic mean of adults shows that mid-life adults are slightly more trusting than younger and older adults. Mid-life adults appeared to be the most likely to agree to participate in international databases and to be informed of secondary findings, while older adults were the least likely and the difference was statistically significant. The reason for this interesting result is difficult to explain at this stage and requires further analysis. Eldery patients are more likely to have physicaly disabilities, such as hearing loss and cognitive impairment which reduce their ability to communicate and understand information provided at genetic counseling. There might also be additional psycho-social factors which can prevent older patients form making confident and autonomous decisions regarding genetic testing [13]. Younger respondents on the other hand are slightly more informed of privacy and data protection issues, especially after the implementation of the GDPR legislation across the EU.

Patients without oncological disease were more likely to agree to be included in international databases than patients with a confirmed oncological diagnosis. However, there were no differences regarding other statements. The proportion of explained variance is relatively low, suggesting that other factors affect the propensity to consent. Further research should be performed and should also analyse the associations with diagnoses of other chronic diseases.

We have already mentioned some limitations of our research including the non-inclusion of demographic factors, e.g. place of residence, marital status, occupation, level of education, and previous experience filling out consent forms. It could be assumed that patients with higher education and those living in urban areas would be more inclined to give consent (as well the two factors could be in interaction). People in a relationship (whether married or not married) might be more in favour of giving consent owing to possible benefits of these data for their families or families of other people in a similar situation. In the future, we should repeat the survey with a larger sample and add the aforementioned demographic information to the survey questions. It would also be interesting to know whether the clinical geneticist or genetic counsellor influences the difference in responses, the length of the genetic counselling, and whether informed consent depends on who referred them (self- referral, their doctor, or a relative).

Previous research shows that a number of different factors can affect patient decisions regarding informed consent. For example, a study conducted in southern Ethiopia on the process of obtaining informed consent for biomedical research has shown that the completion of the consent form was largely influenced by the cultural practices, traditions, and beliefs of the patients who wanted to talk to other family members before signing the consent form [14]. Also, a study on the understanding of informed consent was conducted in Nigeria, showing that almost half of the married women wanted to talk to their husbands before giving consent [15].

It would be advisable to consider sending the consent form to the tested subject (by post) before their genetic counselling appointment. This would give them time to prepare for the questions, talk to the family, and ask about any uncertainties during the consultation. Our patients are often pressed for time as they have other appointments at the IOL or elsewhere. The benefit of providing the consent form in advance would be greater preparedness on the patients side. Paradoxically, such an approach could also lead to greater levels of confusion in patients misinformed by their relatives and friends prior to genetic consultation. Implementation of such an approach should therefore be undertaken with caution as it might prove counterproductive and may lead to longer counselling sessions.

Some researchers, e.g. Bunnik et al. [16] question what oncologists can learn from the consent form developed by clinical geneticists, particularly the importance of explaining the possible results of genetic testing and patients’ expectations. In 2017, Samuel et al. [7] published a study assessing how much effort healthcare professionals put into patients’ understanding of the consent form in clinical genetics based on in-depth interviews and focus groups. In addition, Fowler et al. [4] looked at differences between consent forms from eighteen laboratories. They concluded that standardisation of the informed consent form and its consistent use might help to improve doctor-patient communication and increase understanding of the importance of genetic testing.

## Conclusion

The present study is the first in Slovenia to examine the decisions made by patients before cancer genetic testing, as recorded in their informed consent forms. We were interested in how patients make decisions regarding the storage of biological material, participation in international databases, reporting secondary findings, and communicating results to their relatives. Considerable time is spent on these topics in the pre-genetic consultation, and this area has stringent legislation.

Considering the survey results, it can be stated with certainty that the patients are well informed about the consent form. In addition to explaining the procedure, the clinical geneticist reiterates the most important information to the patient when completing the form.

Implementing the GDPR in the European Union [17] has also made involving individuals in domestic and foreign research much more challenging. It requires a lengthy consent procedure, and the forms used by domestic and foreign research institutions are complex, lengthy and often incomprehensible to patients. It is questionable whether patients even read and understand them thoroughly.

Nevertheless, as our survey shows, most patients are willing (more than 98%) to participate in international databases. In addition, during the consultation, some oncology patients emphasise their wish to contribute to better treatment by participating in cancer research in the future. It is, therefore, essential that, while adequately protecting the subject’s interests, we do not make it difficult for them to participate in international databases projects by providing inadequate information or inappropriately drafted consent forms.

As laws, regulations, conventions, ethical principles, and the profession change over time, so will the informed consent form. Therefore, regardless of whether or not the number of statements to which the subject is asked to consent increases, the clinical geneticist or genetic counsellor must always be professional and well prepared to the extent that he/she can explain the meaning of genetic testing and the completion of consent to the subject in the most appropriate and non-judgemental way possible.

In a future study, it would be helpful to explore the area of informed consent in more depth using qualitative methods. In addition to those who consented to genetic analysis, patients who refused to consent should also be included. Semi-structured interviews could be used to understand the problem’s complexity. This method is probably more suitable than focus groups, since the topic is highly sensitive and very personal and patients might be reluctant to share their views in a group setting. We would be interested in their concerns, attitudes, opinions, values, motivations and possibly ethical, moral or cultural differences. It would be valuable to see, what reasons patients have either to consent or refuse sharing their data.

## Data Availability

No datasets were generated or analysed during the current study.
